# Protective effects of ulinastatin and methylprednisolone against radiation-induced lung injury in mice

**DOI:** 10.1093/jrr/rrw036

**Published:** 2016-09-30

**Authors:** Yu Sun, Yu-Jun Du, Hui Zhao, Guo-Xing Zhang, Ni Sun, Xiu-Jiang Li

**Affiliations:** 1 Intensive Care Unit, Cancer Hospital of Jilin Province, Changchun 130021, China; 2 Department of Nephrology, Bethune First Hospital of Jilin University, Changchun 130021, China

**Keywords:** ulinastatin, methylprednisolone, radiation-induced lung injury, transforming growth factor β1 (TGF-β1), tumor necrosis factor α (TNF-α)

## Abstract

The effectiveness of ulinastatin and methylprednisolone in treating pathological changes in mice with radiation-induced lung injury (RILI) was evaluated. Forty C57BL/6 female mice received whole-chest radiation (1.5 Gy/min for 12 min) and were randomly allocated into Group R (single radiation, *n =* 10), Group U (ulinastatin treatment, *n =* 10), Group M (methylprednisolone treatment, *n =* 10), or Group UM (ulinastatin and methylprednisolone treatment, *n =* 10). Another 10 untreated mice served as controls (Group C). Pathological changes in lung tissue, pulmonary interstitial area density (PIAD) and expression levels of transforming growth factor β1 (TGF-β1) and tumor necrosis factor α (TNF-α) in lung tissue, serum and bronchoalveolar lavage fluid were determined. Alleviation of pathological changes in lung tissue was observed in Groups U, M and UM. Treatment with ulinastatin, methylprednisolone or both effectively delayed the development of fibrosis at 12 weeks after radiation. Ulinastatin, methylprednisolone or both could alleviate the radiation-induced increase in the PIAD (*P* < 0.05 or *P* < 0.01). Treatment with ulinastatin, methylprednisolone or both significantly reduced the expression of TNF-α, but not TGF-β1, at 9 weeks after radiation compared with Group R (*P* < 0.01). Ulinastatin and**/**or methylprednisolone effectively decreased the level of TNF-α in lung tissue after RILI and inhibited both the inflammatory response and the development of fibrosis.

## INTRODUCTION

Radiation-induced lung injury (RILI) is a common and severe complication in patients undergoing thoracic radiotherapy, receiving bone marrow transplantation, or involved in nuclear accidents [1–3]. Its damage to normal tissue is seen increasingly with the ever more widespread application of radiotherapy for the treatment of tumors. Radiation pneumopathy is a response of various target cells in normal lung tissue to radiation [4]. The early phase after acute lung damage is characterized by exudative inflammation complicated by interstitial pneumonia 6–12 weeks after exposure. A later phase is characterized by chronic inflammation, tissue remodeling, and pulmonary fibrosis [5].

Inflammatory cytokines, including transforming growth factor β1 (TGF-β1) and tumor necrosis factor α (TNF-α), play critical roles in regulating the RILI process [6]. TNF-α is involved in the initiation and amplification of the inflammatory responses during RILI [7], and TGF-β1 mediates the proliferation and differentiation of fibroblasts into myofibroblasts, which play a critical role in the fibrotic process via the production of collagen and other extracellular matrix proteins [8].

Ulinastatin is a broad-spectrum protease inhibitor that inhibits hydrolase activity and has protective roles in multiple organs [9]. Ulinastatin can alleviate ventilator-related lung injury and the acute lung injury induced by lipopolysaccharide [10, 11]. Bao *et al*. [7] reported the effect of ulinastatin in patients with non–small cell lung cancer after radiation therapy, and their results indicated that the incidence and grade of RILI, as well as the reduction of pulmonary function, were significantly lower in patients treated with ulinastatin than in controls. In addition, the administration of ulinastatin during the post-radiation period suppresses TGF-β expression and lung fibrosis, resulting in significantly prolonged survival of irradiated mice [12]. In the late 1980s, Wesselius *et al*. [13] demonstrated that the number of inflammatory cells is significantly diminished during acute RILI after corticosteroid treatment. Methylprednisolone could relieve lipopolysaccharide (LPS)-induced acute lung injury [14], but a prolonged high dose also has harmful effects during this treatment [15]. Yang *et al*. [16] reported that methylprednisolone combined with bone marrow mesenchymal stem cells can inhibit paraquat-induced acute lung injury. The combined use of methylprednisolone and ulinastatin has a major effect on tumor cell metastasis after surgical stress and experimental autoimmune encephalomyelitis [17, 18]. However, there has been no report concerning combined therapy with ulinastatin and methylprednisolone in RILI.

The present study was designed to evaluate the effectiveness of ulinastatin and methylprednisolone for the treatment of pathological changes in a mouse model of RILI.

## MATERIALS AND METHODS

### Materials

A total of 50 C57BL/6 female mice (20 ± 2 g) were purchased from the Experimental Animal Center of the Department of Basic Medicine of our University (China). An animal X-ray irradiator (X-RAD 320) was obtained from PXi (Precision X-ray, USA). A microscopic system (OLYMPUS-BX-51, Japan) was used to perform image acquisition, and Image-Pro Plus 6.0 software (Media Cybernetics, USA) was used to analyze the images. A microplate reader (Multiskan K3, Thermo, USA) was used to determine the levels of TGF-β1 and TNF-α via enzyme-linked immunosorbent assay (ELISA) kits (Sigma-Aldrich, USA). Immunohistochemistry kits were also purchased from Sigma-Aldrich. Ulinastatin was purchased from Techpool (Guangzhou, China).

This study was approved by the Medical Ethics Committee of the Department of Basic Medicine of our University (China).

### Establishment of animal model

The mice were anesthetized by intraperitoneal injection of pentobarbital (60 mg/kg). The onset time of anesthesia was 2–5 min, and the average recovery time was 36 min. Forty mice were fixed and placed in the supine position for radiation. The chest of the mice was covered with a 23-mm-thick wax block to control the dose of radiation, and the head and the abdomen were shielded by lead plates. The mice were placed 1 m from the radiation source, and the exposure field was 1 × 3 cm. The whole thoracic radiation was performed with a single dose of 18 Gy (1.5 Gy/min for 12 min). The mice were randomly allocated into Group R (single radiation, *n =* 10), Group U (intraperitoneal injection of 400 000 U/kg·d ulinastatin for 7 d, *n =* 10), Group M (intraperitoneal injection of 40 mg/kg·d methylprednisolone for 7 d, *n =* 10) or Group UM (intraperitoneal injection of 400 000 U/kg·d ulinastatin and 40 mg/kg·d methylprednisolone for 7 d, *n =* 10). In Group UM, methylprednisolone and ulinastatin were administered as a combined injection in 1 ml of 0.9% sodium chloride solution, and this volume was consistent with that used in Group U and Group M. The other 10 untreated mice served as controls (Group C). The mice in Groups C and R received an equal volume of normal saline simultaneously. All mice were injected from the day following radiation.

### Collection of specimens

Two mice from each group were sacrificed at 2, 6, 9, 12 and 15 weeks after radiation for collection of blood and other samples. The blood samples were centrifuged at 1200 *g* and 4°C for 20 min, and the serum was separated and kept at −80°C for tests. The mice underwent pneumonectomy, and the bronchoalveolar lavage fluid (BALF) was collected by repeated lavage for three times with 1 ml normal saline through the main bronchus of the left lung. The BALF was centrifuged at 700 *g* and 4°C for 10 min to separate the supernatant, which was then kept at −80°C for analysis. The right lower lobe was fixed in 10% paraformaldehyde, embedded in paraffin, and then sectioned for immunohistochemical analysis.

### Pathological examination of lung tissue

Pathological changes in the lung tissue in each group were examined after hematoxylin/eosin (HE) staining. A total of 10 fields in each section were randomly chosen for statistical analysis. Image Pro-Plus 6.0 software (Media Cybernetics, USA) was used to analyze the pulmonary interstitial area density (PIAD), which was the pulmonary interstitial area per × 200 field.

### Immunohistochemistry

The expression levels of TGF-β1 and TNF-α in lung tissue were observed by immunohistochemical staining. The integral optical density (IOD) was determined across a 400 × field, using Image Pro-Plus 6.0 software to reflect the protein expression. A total of 10 fields in each section were randomly chosen for statistical analysis.

### ELISA

At 9 weeks after irradiation, the concentrations of TGF-β1 and TNF-α in serum and BALF were determined by ELISAs.

### Statistical analysis

SPSS (19.0) software was used for statistical analysis. The data distribution was determined using the Shapiro–Wilk test. If normally distributed, the data were presented as mean ± standard deviation (SD), and groups were compared using analysis of variance (ANOVA). *Post-hoc* tests were performed if a statistical significance was found by ANOVA. Differences with a *P*-value <0.05 were considered statistically significant.

## RESULTS

### Pathological changes in lung tissue

**Fig. 1.**
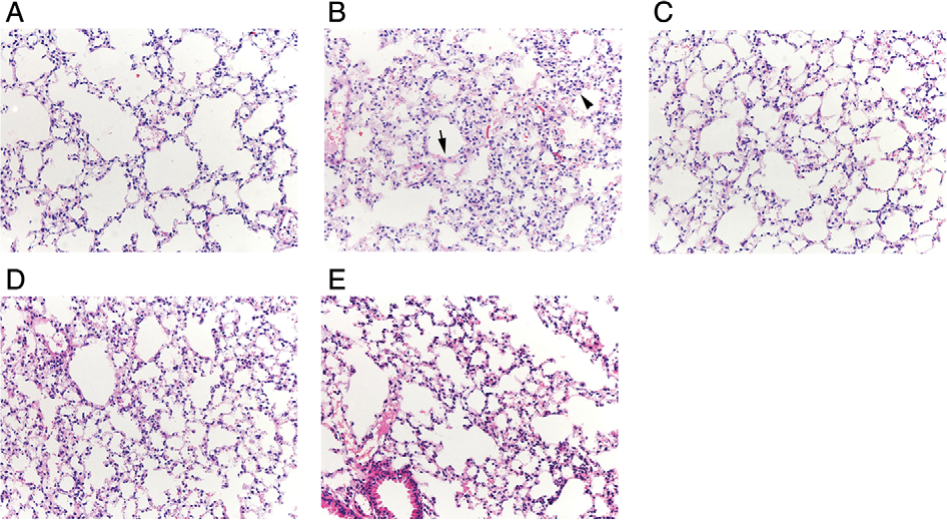
Pathological changes in lung tissue at 2 weeks after radiation in each group. HE staining showed that the normal structure of lung tissue was observed in Group C (A). Thickened alveolar walls (arrow), pulmonary capillary expansion, and hyperemia and inflammatory infiltration foci (arrowhead) were found in Group R (B). Alleviation of these pathological changes was observed in Groups U, M and UM (C–E).

**Fig. 2.**
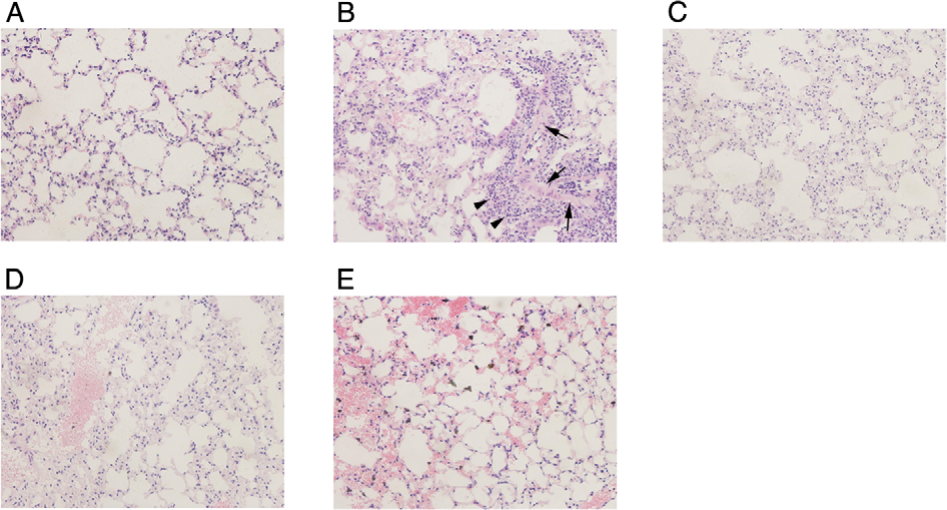
Pathological changes in lung tissue at 12 weeks after radiation in each group. Compared with normal lung tissue (A), pulmonary fibrotic changes (arrow), and infiltration of inflammatory cells (arrowhead) were observed at 12 weeks after radiation (B). Treatment with ulinastatin, methylprednisolone or both effectively delayed the development of fibrosis and inhibited infiltration of inflammatory cells (C–E).

The results of HE staining showed that the lung tissue in Group C had normal structure (Fig. 1A). However, thickened alveolar walls, pulmonary capillary expansion, and hyperemia and inflammatory infiltration foci were observed 2 weeks after radiation in Group R (Fig. 1B). Alleviation of these pathological changes was observed in Groups U, M and UM, suggesting that ulinastatin and methylprednisolone had protective effects during the early phase after radiation exposure (Fig. 1C–E). Pulmonary edema and localized lung consolidation appeared at 6 weeks, and pulmonary fibrotic changes were observed 12 weeks after radiation (Fig. 2B). Treatment with ulinastatin, methylprednisolone or both effectively delayed the development of fibrosis and inhibited the infiltration of inflammatory cells (Fig. 2C–E).

**Fig. 3.**
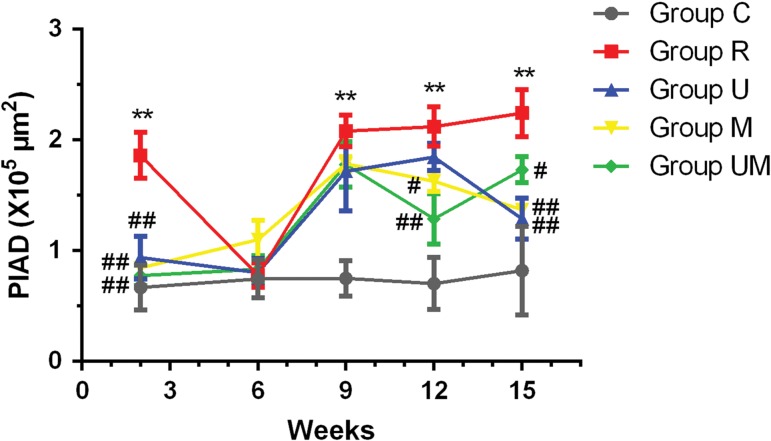
Dynamic changes in the PIAD in each group. The PIAD was significantly greater in Group R than in Group C at 2 weeks after radiation (*P* < 0.01), but significantly lower in Groups U, M and UM compared with that in Group R (all *P* < 0.01). No statistically significant differences were found between the groups at 6 weeks after radiation (*P* > 0.05). At 9 weeks after radiation exposure, the PIAD was greater in Groups R, U, M and UM than in Group C (all *P* < 0.01), but no statistically significant differences were found between these groups (*P* > 0.05). A significant difference emerged between Group M or UM and Group R at 12 weeks (*P* < 0.05 and *P* < 0.01, respectively), which predated the statistically significant difference observed between Group U and Group R at 15 weeks (*P* < 0.01). In addition, the statistically significant differences in the PIAD were still observed between Group M or UM and Group R (*P* < 0.01 and *P* < 0.05, respectively). ^**^P < 0.01 vs Group C; ^#^*P* < 0.05, ^##^*P* < 0.01 vs Group R.

The PIAD was dynamically determined to evaluate the pathological changes in the lung tissue after radiation (Fig. 3). The PIAD was significantly greater in Group R than in Group C at 2 weeks after radiation (*P* < 0.01), and this value was significantly lower in Groups U, M and UM compared with that in Group R (all *P* < 0.01). However, no statistically significant differences were found between the groups at 6 weeks after radiation (*P* > 0.05). At 9 weeks after radiation exposure, the PIAD was observed to be greater in Groups R, U, M and UM compared with that in Group C (all *P* < 0.01), but no statistically significant differences were found between these groups (*P* > 0.05). A significant difference emerged between Group M or UM and Group R at 12 weeks (*P* < 0.05 and *P* < 0.01, respectively), which occurred sooner than the statistically significant difference that emerged between Groups U and R at 15 weeks (*P* < 0.01). In addition, statistically significant differences in the PIAD were still observed between Group M or UM and Group R (*P* < 0.01 and *P* < 0.05, respectively, Fig. 3).

### Expression of TGF-β1 and TNF-α in lung tissue

**Fig. 4.**
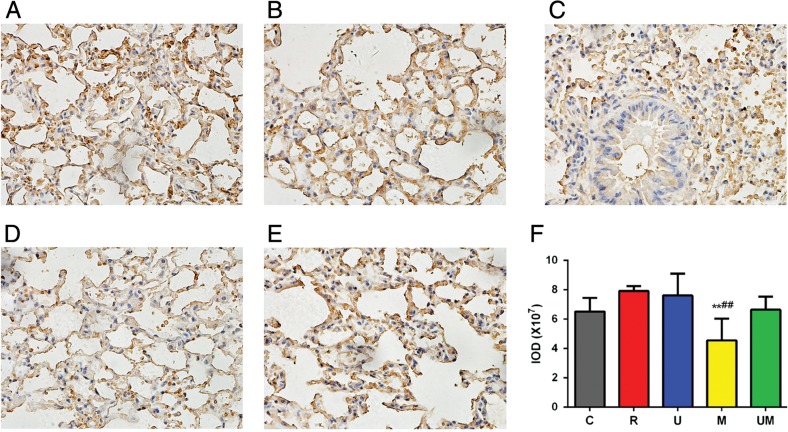
Expression of TGF-β1 in lung tissue at 9 weeks after radiation in each group. TGF-β1 expression was evaluated in the alveolar interval, bronchioli smooth muscle, vascular smooth muscle, vascular endothelium, and perivascular space. The expression levels in these areas were not statistically different between Groups C, R, U, and UM (A, B, C and E). TGF-β1 expression was significantly downregulated at 9 weeks in Group M compared with levels in Groups C and R (both *P* < 0.01, D and F).

**Fig. 5.**
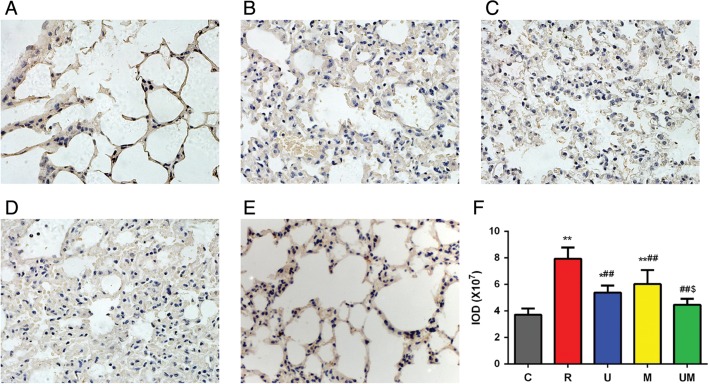
Expression of TNF-α in lung tissue at 9 weeks after radiation in each group. TNF-α expression was significantly greater in Group R than in Group C (*P* < 0.01, A, B, C and F). Treatment with ulinastatin, methylprednisolone or both significantly reduced the expression of TNF-α compared with that in Group R (all *P* < 0.01, B, C, D, E and F). Furthermore, the expression of TNF-α in Group UM was significantly lower than that in Group U (*P* < 0.05, C, E and F).

To determine whether inflammatory factors play a critical role in the changes in lung tissue after radiation and corresponding treatments, we compared the expression levels of TGF-β1 and TNF-α among the groups at 9 weeks after radiation exposure. TGF-β1 expression was observed in the alveolar interval, bronchioli smooth muscle, vascular smooth muscle, vascular endothelium, and perivascular space. The expression levels in these areas did not differ significantly between Groups C, R, U and UM (Fig. 4A, B, C and E). However, TGF-β1 expression was significantly downregulated at 9 weeks in Group M compared with levels in Groups C and R (both *P* < 0.01, Fig. 4D and F). TNF-α expression was significantly greater in Group R than in Group C (*P* < 0.01, Fig. 5A, B, C and F). Treatment with ulinastatin, methylprednisolone or both significantly reduced the expression of TNF-α compared with the level in Group R (all *P* < 0.01, Fig. 5B, C, D, E and F). Furthermore, the expression of TNF-α in Group UM was significantly lower than that in Group U (*P* < 0.05, Fig. 5C, E and F).

### Levels of TGF-β1 and TNF-α in serum and BALF

**Fig. 6.**
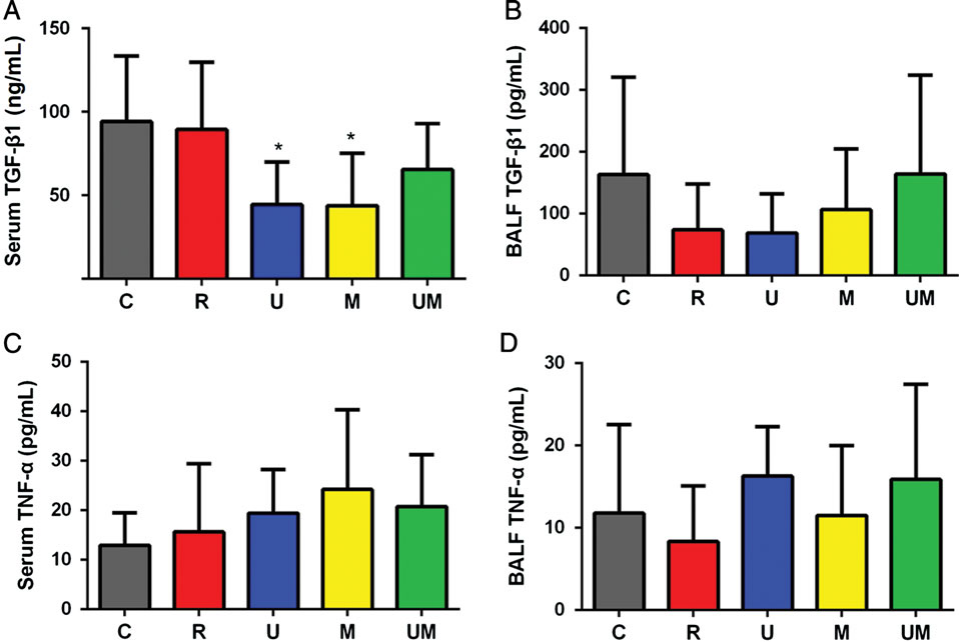
Comparison of serum and BALF levels of TGF-β1 and TNF-α in each group. The serum level of TGF-β1 was significantly lower in Groups U and M than in Group C (both *P* < 0.05, A). However, there were no statistically significant differences in the BALF levels of TGF-β1 between the groups (*P* > 0.05, B). In addition, no statistically significant differences were found in either the serum or BALF levels of TNF-α between the groups (all *P* > 0.05, C–D).

We compared the levels of TGF-β1 and TNF-α in serum and BALF using ELISAs. Previous study showed that irradiation-mediated TNF-α release was significantly increased and reached maximal values at 8 weeks post-irradiation [19]. Thus, we compared the levels of cytokines among groups, with more emphasis on Week 9. The serum levels of TGF-β1 were significantly lower in Groups U and M than in Group C (both *P* < 0.05, Fig. 6A), whereas no significant differences in the BALF level of TGF-β1 were observed among the groups (*P* > 0.05, Fig. 6B). In addition, the serum and BALF levels of TNF-α did not differ significantly among the groups (all *P* > 0.05, Fig. 6C and D).

## DISCUSSION

RILI is caused by multiple etiologies, which involve both radiation injury to various types of target cells and the release of multiple cytokines by these target cells and their related cells [20]. With the growth of granulation and tissue repair, pulmonary interstitial fibrosis occurs in the later phase of RILI, following the proliferation of fibroblasts and the production of collagen, resulting in respiratory failure of the patients, which is one of the leading causes of death after RILI. Supportive treatment, mobilization of airway secretions, anti-inflammatory treatment, and control of acute exacerbation are common therapy options [21]. Appropriate medications mainly include glucocorticoids, non-steroidal anti-inflammatory drugs, and γ-interferon, and drugs that reduce lung injury serve as adjuvants. Inflammation in the early period can be controlled using these medications. However, considerable pulmonary interstitial fibrosis develops in the later stage, and the treatment remains unsatisfactory.

In this study, an acute reaction of the lung tissue, including thickened alveolar walls, pulmonary capillary expansion, and hyperemia and inflammatory infiltration foci, were found 2 weeks after radiation in Group R. In contrast, alleviation of these pathological changes was observed in Groups U, M and UM, suggesting that ulinastatin and methylprednisolone had protective effects during the early phase after radiation exposure. Treatment with ulinastatin, methylprednisolone or both effectively delayed the development of fibrosis at 12 weeks after radiation. The dynamic examination of PIAD suggested that ulinastatin, methylprednisolone or both could alleviate the radiation-induced increase in the PIAD at an early stage after RILI. Furthermore, these data revealed a protective effect of ulinastatin, methylprednisolone or both against the development of fibrosis in the later stage of RILI.

TGF-β1, a growth regulatory factor originating from multiple human epithelia, has various biological effects related to cellular proliferation and differentiation, deposition of extracellular matrix, and human immune responses [22]. TGF-β1 is a key cytokine that regulates cellular growth and controls the balance of extracellular matrix production and degradation [23]. Fibroblasts, macrophages and alveolar epithelial cells are effectors of TGF-β1 in radiation-induced fibrosis [24]. Pulmonary fibrosis that appears at 15 weeks after radiotherapy is correlated with the level of TGF-β1 [25]. Treatment with ulinastatin inhibited the expression of TGF-β1 at 15 weeks post radiation [26]. In this study, we did not observe an inhibition of TGF-β1 expression at 9 weeks after radiation, which was associated with a change in the PIAD. However, treatment with methylprednisolone did downregulate the expression of TGF-β1 at 9 weeks after radiation, suggesting that methylprednisolone mediates earlier effects than ulinastatin to protect the lung tissue.

TNF-α is considered the most relevant factor associated with the development of radiation-induced pulmonary fibrosis, and TNF-α plays a critical role in the occurrence and development of radiation pneumonitis [27, 28]. In this study, treatment with ulinastatin, methylprednisolone or both significantly reduced the expression of TNF-α at 9 weeks after radiation compared with Group R, suggesting that ulinastatin and methylprednisolone protected lung tissue via inhibition of the TNF-α–related signaling pathway. Interestingly, in this study, the therapeutic effects of ulinastatin and methylprednisolone on RILI were not associated with changes in the serum or BALF levels of TGF-β1 and TNF-α.

These data suggest that radiation pneumonitis develops gradually and is associated with the level of TNF-α in lung tissue. The mice treated with ulinastatin, methylprednisolone or both showed significantly alleviated pulmonary edema and inflammation of the alveoli compared with those that received a single radiation exposure. In addition, these treatments could alleviate fibrosis in the lung. However, we did not observe an obvious benefit in the combination group (UM) compared with the individual treatments (groups U or M)—this result was not expected—thus, the underlying mechanism remains unknown. Other inflammatory cytokines are also involved in the pathological changes of RILI. The roles of interleukin 6 (IL-6), CD36, and the chemokine series in RILI should be evaluated in future studies.

Ulinastatin and/or methylprednisolone effectively decreased the level of TNF-α in lung tissue after RILI and inhibited the inflammatory response and the development of fibrosis. These findings expand the current therapy regimens for RILI and provide a new choice for preventing and treating lung injury and fibrosis in patients undergoing thoracic radiotherapy, receiving bone marrow transplantation, or affected by nuclear accidents.

## FUNDING

This work was supported by a research grant from the Science and Technology Bureau of Changchun City [No. 12SF61].

## CONFLICT OF INTEREST

The authors declare that there are no conflicts of interest.
